# Detection of Δ^9^ THC in oral fluid following vaporized cannabis with varied cannabidiol (CBD) content: An evaluation of two point‐of‐collection testing devices

**DOI:** 10.1002/dta.2687

**Published:** 2019-09-10

**Authors:** Thomas R. Arkell, Richard C. Kevin, Jordyn Stuart, Nicholas Lintzeris, Paul S. Haber, Johannes G. Ramaekers, Iain S. McGregor

**Affiliations:** ^1^ Lambert Initiative for Cannabinoid Therapeutics The University of Sydney Sydney New South Wales Australia; ^2^ Brain and Mind Centre The University of Sydney Sydney New South Wales Australia; ^3^ Faculty of Medicine, Central Clinical School The University of Sydney Sydney New South Wales Australia; ^4^ Faculty of Science, School of Psychology, Brain and Mind Centre The University of Sydney Sydney New South Wales Australia; ^5^ The Langton Centre, Drug and Alcohol Services South East Sydney Local Health District, NSW Health New South Wales Australia; ^6^ Drug Health Services Royal Prince Alfred Hospital Camperdown New South Wales Australia; ^7^ Faculty of Psychology and Neuroscience Maastricht University The Netherlands

**Keywords:** cannabis, CBD, oral fluid, point‐of‐collection testing, THC

## Abstract

Point‐of‐collection testing (POCT) for Δ^9^‐tetrahydrocannabinol (THC) in oral fluid is increasingly used to detect driving under the influence of cannabis (DUIC). However, previous studies have questioned the reliability and accuracy of two commonly used POCT devices, the Securetec DrugWipe^®^ 5 s (DW5s) and Dräger DrugTest^®^ 5000 (DT5000). In the current placebo controlled, double‐blind, crossover study we used liquid chromatography‐tandem mass spectrometry (LC–MS/MS) to accurately quantify cannabinoid concentrations in the oral fluid of 14 participants at various timepoints (10, 60, 120, and 180 minutes) following vaporization of 125 mg of THC‐dominant (11% THC; <1% CBD), THC/CBD equivalent (11% THC; 11% CBD) and placebo (<1% THC; <1% CBD) cannabis. At each timepoint, oral fluid was also screened using the DW5s (10 ng/mL THC cut‐off) and DT5000 (10 ng/mL THC cut‐off). LC–MS/MS analysis showed peak oral fluid THC concentrations at the 10 minute timepoint with a rapid decline thereafter. This trajectory did not differ with THC dominant and THC/CBD equivalent cannabis. With a 10 ng/mL confirmatory cut‐off, 5% of DW5s test results were false positives and 16% false negatives. For the DT5000, 10% of test results were false positives and 9% false negatives. Neither the DW5s nor the DT5000 demonstrated the recommended >80% sensitivity, specificity and accuracy. Accuracy was lowest at 60 minutes, when THC concentrations were often close to the screening cut‐off (10 ng/mL). POCT devices can be useful tools in detecting recent cannabis use; however, limitations should be noted, and confirmatory LC–MS/MS quantification of results is strongly advisable.

## Graphical abstract

The ongoing legalization of medical and recreational cannabis presents new challenges for law enforcement and traffic safety regulations. Here, oral fluid THC and CBD concentrations were quantified via LC–MS/MS and the performance of two point‐of‐collection testing (POCT) devices was assessed following controlled vaporization of cannabis with varied CBD content. POCT can detect very recent cannabis use, but there are concerns around reliability and accuracy when these devices are used in real‐world settings.

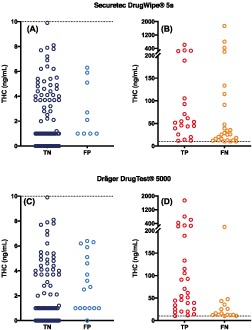

## 1 INTRODUCTION

The ongoing amendment of medicinal and recreational cannabis laws worldwide has made driving under the influence of cannabis (DUIC) a key public safety concern.^1^, ^2^ There are two main approaches that are used to assess DUIC. The first is an effect‐based approach whereby a police officer or drug recognition expert (DRE) must demonstrate behavioral impairment. Although widely used, there are concerns around the effectiveness of this approach and cases can be difficult to prosecute.^3^, ^4^ Many jurisdictions therefore enforce per se or zero tolerance policies for DUIC. Under such laws, a driver has committed an offence if delta‐9‐tetrahydrocannabinol (THC) is detected at or above a given concentration in a specified biological matrix, irrespective of actual impairment. Oral fluid is now increasingly relied upon as a matrix for DUIC detection as samples can be readily obtained in a non‐invasive manner and rapidly analyzed at the roadside using point‐of‐collection testing (POCT) devices.

POCT devices are used by authorities to detect DUIC in a number of countries including Norway,^5^ Germany,^6^ Belgium,^7^ and Australia, where this process is referred to as mobile drug testing (MDT). First introduced in 2004 in the state of Victoria, Australia,^8^ MDT has since been adopted by all Australian states and territories. In the state of New South Wales (NSW), Australia, the procedure involves an initial test for oral fluid THC using the Securetec DrugWipe^®^ (DW) device. If positive, this is followed by a secondary test using the Dräger DrugTest^®^ 5000 (DT5000) device. If the DT5000 test is also positive, confirmatory analysis is conducted to confirm the presence of THC. Authorities have not revealed the THC screening cut‐offs used for MDT: the DT5000 screening cut‐off can be set by the operator to 5, 10, or 25 ng/mL, and the DW device variant which is used by NSW Police is not commercially available.

The primary aim of MDT is to improve road safety by detecting and therefore deterring DUIC. It is therefore essential that POCT devices accurately discriminate between drug‐positive and drug‐negative cases. False positives may lead to unjust punishment for drivers (eg, license disqualification, criminal conviction), while false negatives undermine the aims and integrity of the MDT program. To assess the real‐world performance of the DW5s and DT5000 devices, controlled laboratory studies that compare POCT device results with confirmatory analysis using sensitive analytical methods are crucial.

A major EU study of drug‐affected driving (DRUID) recommended that POCT devices demonstrate a minimum 80% sensitivity (the ability to correctly detect drug‐positive samples), specificity (the ability to correctly determine drug‐negative samples), and accuracy (the ability to differentiate drug‐positive from drug‐negative samples).^9^ In prior studies involving earlier versions of the DW devices (various models; 20–30 ng/mL THC cut‐off), sensitivity, specificity, and accuracy were variously reported as 22%–89.1%, 50%–100%, and 53%–94%.^9^, ^10^, ^11^, ^12^, ^13^, ^14^ A study using a more recent version of the DW (DW5s; 15 ng/mL THC cut‐off) reported sensitivity, specificity and accuracy as 51%, 100%, and 68% relative to a 10 ng/mL confirmatory cut‐off.^15^ For the DT5000 (5–25 ng/mL THC cut‐off), performance, sensitivity, specificity and accuracy were variously reported as 49.5%–100%, 55%–90%, and 55%–86.4%.^5^, ^9^, ^14^, ^16^, ^17^, ^18^, ^19^, ^20^ The high variability in these results reflects differences in the cut‐offs and biological matrices used for confirmatory analyses, the timing of tests relative to cannabis administration, cannabis dosage, and route of administration.

Vaporization of cannabis is an increasingly common route of administration among both medicinal and recreational cannabis users;^21^, ^22^, ^23^ however, only a small number of studies have described oral fluid concentrations^24^, ^25^ and POCT device performance^20^ following vaporized cannabis. Moreover, these studies have been limited to THC‐dominant cannabis. Cannabis chemovars (‘strains') and medicinal cannabis products often contain significant levels of cannabidiol (CBD), a non‐intoxicating cannabinoid with anxiolytic, anticonvulsant, and antipsychotic properties.^26^, ^27^, ^28^ For example, the ‘light cannabis' products that are legally available in a number of EU countries must contain less than 0.2% THC but may contain up to 40% CBD.^29^ Medicinal cannabis products containing both THC and CBD include Nabiximols (Sativex), a buccal spray with a 1:1 ratio of THC and CBD, as well as commercially available cannabis botanicals and extracts^30^ and homegrown illicit artisanal preparations.^31^ It is currently unclear whether CBD content might influence the performance of POCT devices or influence the underlying pharmacokinetics of THC in oral fluid.

The current study therefore sought to evaluate the performance of the DW5s and DT5000 POCT devices relative to liquid chromatography−tandem mass spectrometry (LC–MS/MS) confirmatory analysis following controlled laboratory vaporization of THC‐dominant (11% THC, <1% CBD [hereafter ‘THC']); THC/CBD equivalent (11% THC, 11% CBD [hereafter ‘THC/CBD']) and placebo (<1% THC; <1% CBD) cannabis using a double‐blind, within‐subjects, crossover design. This occurred as part of larger study examining the effects of THC‐dominant and THC/CBD‐equivalent cannabis on driving and cognition that has been published elsewhere.^32^

## 2 METHODS

### 2.1 Participants

Healthy adults (aged 18–65 years) with a history of infrequent cannabis use were recruited for this study. Inclusion criteria were self‐reported cannabis consumption ≤2 times/week in the previous three months and ≥ 10 lifetime exposures (see Table 1 for details). Exclusion criteria included current mood disorder; lifetime major psychiatric illness; history of clinically significant adverse response to previous cannabis exposure; any moderate or severe substance use disorder as assessed by an addiction medicine specialist; pregnant/nursing; interest in treatment to reduce cannabis use; current use of medications known to affect driving; active hypertension, cardiovascular disease, or chronic pulmonary disease. Volunteers were recruited through online advertisement, social media (e.g., Facebook) and word of mouth. All participants meeting inclusion/exclusion criteria underwent a comprehensive medical and psychiatric evaluation and provided written informed consent prior to study enrolment. All procedures were approved by the Sydney Local Health District (RPAH Zone) Human Research Ethics Committee. The trial was listed on the Australia New Zealand Clinical Trials Registry (No. 12616000414415).

**Table 1:** Participant characteristics and cannabis use history

Subject	Age (years)	Gender	BMI (kg/m^2^)	Highest Level of Education Completed	AUDIT‐C Score	Age at First Cannabis Use	Days Since Last Cannabis Use and Study Enrolment (#)	Days of Cannabis Use in Last Month (#)	Hours Stoned on a Typical Occasion (#)
1	21	M	24.9	HS	6	16	12	4	4
2	24	F	24.64	HS	7	16	2	4	2
3	38	F	23.74	T	6	16	22	2	3
4	31	M	26.2	HS	4	18	163	0	6
5	24	M	20.74	T	4	17	1	13	6
7	24	M	40	T	6	17	57	0	5
9	27	M	27	HS	4	25	71	0	4
11	29	M	23.8	T	6	15	4	8	3
12	24	F	30.53	T	5	21	101	0	3
13	30	M	21.3	T	5	15	3	12	2
14	26	M	23	T	4	18	5	5	3
15	26	M	21.69	T	3	18	5	7	4
16	28	M	22.7	T	7	19	1	14	2
17	33	M	26.8	HS	7	19	2	4	8
Mean	27.5		25.50		5.29	17.86	32.07	5.21	3.93
SD	4.29		4.75		1.33	2.56	47.34	4.77	1.71

M = male; F = female; HS = high school; T = tertiary

### 2.2 Study design and procedures

This randomized, placebocontrolled, within‐subjects, double‐blind, crossover study included three experimental sessions that were scheduled at least seven days apart to avoid carryover effects. Participants were instructed to abstain from illicit drugs for the duration of the study (i.e., from the time of study enrolment until the final session) and from alcohol on the night before research sessions, to maintain any use of regular medications, and to consume no more than their regular caffeine intake on the morning of research sessions. Participants arrived at the clinical research unit at 9 am on the morning of research sessions. Zero breath alcohol concentration (BrAC) was confirmed via breathalyzer (Alcotest 5510, Draeger, Lübeck, Germany) and participants were initially screened using the DrugWipe^®^ 5 s to rule out acute drug intoxication and/or recent drug use. Participants testing positive for any drug (cannabis, amphetamine, methamphetamine, cocaine, or opiates) were sent home and the session was rescheduled.

Participants inhaled 125 mg THC‐dominant (‘THC'; 11% THC; <1% CBD), THC/CBD equivalent (‘THC/CBD'; 11% THC, 11% CBD) or placebo (<1% THC; <1% CBD) cannabis (Tilray, BC, Canada) via vaporization at 200°C (Mighty Medic, Storz & Bickel, Tuttlingen, Germany), resulting in projected doses of approximately 13.75 mg THC and CBD. Cannabinoid concentrations were determined by Tilray using high‐performance liquid chromatography (HPLC). Vaporization occurred over 5 minutes according to a standardized procedure (inhale 3 seconds, hold 3 seconds, exhale and rest 30 seconds). If vapor was still visible in exhaled breath at 5 minutes, then this procedure was continued until vapor was no longer visible to ensure complete vaporization of plant material. Across three sessions, separated by at least seven days, participants received the three study drugs (one per session) in a randomized and counterbalanced order. The randomization schedule was created by an independent researcher, and only the study pharmacist had access to the randomization code.

### 2.3 Oral fluid collection and POCT procedures

Oral fluid samples were collected using Quantisal™ collection devices (Immunalysis, Pomona, CA, USA) at baseline and at 10, 60, 120, and 180 minutes post‐vaporization. Devices were placed under the tongue until indicators turned blue (collecting 1.0 ± 0.1 mL of oral fluid), or for a maximum of 10 minutes, and placed into the stabilizing buffer. Samples were kept at 4°C until analysis which occurred within a month of collection. Food and drink consumption were disallowed for 10 minutes prior to collection.

Oral fluid tests were also performed at 10, 60, 120, and 180 minutes after vaporization using the Securetec DrugWipe^®^ 5 s (Securetec, Neubiberg, Germany) and Dräger DrugTest^®^ 5000 (Dräger, Lübeck, Germany) devices. Tests were performed in this order immediately following oral fluid sample collection. Both devices had a manufacturer‐specified detection limit of 10 ng/mL THC.

The DW5s test device has two sampling pads which collect oral fluid from the tongue (about 10–20 μL). Participants are instructed to run their tongue around the inside of their mouth in a circular motion three times before slowly scraping the sampling pads down their tongue. Sufficient volume of collected oral fluid is indicated by a change in color of the sampling pads. The researcher then fastens the collection pads to the test strip and breaks an ampoule containing buffer. The test is held vertically for 10 seconds before being laid horizontally and results are visible within 10 minutes. A positive test is indicated by the appearance of a red line. Test results where the DW5s red ‘positive' line was considered too ambiguous were excluded.

The DT5000 test consists of a test cassette, a buffer cartridge, and an analytical instrument. The test cassette comprises a collection pad which collects oral fluid from the cheeks and tongue. Participants are instructed to wipe this pad around the inside of their cheeks and across their gums until sufficient oral fluid has been collected which is indicated by the appearance of a blue line. The test cassette and the buffer cartridge are then inserted into the analyzing instrument. Results are available after 8 minutes (negative, non‐negative, or invalid) and can be printed using an attached printer. Test results where the indicator line did not turn blue were excluded. Test results for both devices were read and filed by an independent observer and only made available to the researchers upon completion of the study.

### 2.4 Oral fluid analysis via LC–MS/MS

Oral fluid samples were analyzed using LC–MS/MS. Duplicate 1 mL aliquots were fortified with an internal standard mixture containing *d*_3_‐THC and *d*_3_‐CBD. Duplicate calibrator samples were prepared using cannabinoid‐free saliva (obtained from healthy volunteers using Quantisal™ collection devices, and checked for cannabinoid content via LC–MS/MS), spiked with THC, CBD, and internal standards to generate a standard curve for each analyte and quality control samples. THC and CBD were isolated using supported liquid extraction (SLE), where each sample aliquot was absorbed onto a 1 mL capacity ISOLUTE^®^ SLE+ column (Biotage, Sydney, Australia), and analytes were eluted with 1.6 mL DCM, 3.5 mL methyl *tert*‐butyl ether (MTBE), and 1.6 mL 1:5 ethyl acetate and MTBE. The eluate was evaporated without heating under a gentle stream of nitrogen, and analytes were reconstituted in 200 μL of 1:1 acetonitrile and 0.1% formic acid in water, transferred to 2 mL autosampler vials fitted with 200 μL capacity glass inserts, and placed in the LC–MS/MS autosampler held at 4°C.

Chromatographic separation was achieved using an Eclipse XDB‐C18 column (50 mm x 2.1 mm i.d., particle size 3.5 μm; Agilent Technologies, Singapore) using gradient elution with mobile phases 0.1% formic acid in water and acetonitrile, at a flow rate of 0.3 mL/min. This was coupled to a Shimadzu LCMS‐8030 mass spectrometer for analyte identification and quantification.

The LC–MS/MS analysis was validated for selectivity, linearity, accuracy, precision, bench‐top and autosampler stability, dilution integrity, limit of detection (LOD), and limit of quantification (LOQ) (Table 2), following Food and Drug Administration (FDA) validation guidelines.^33^ Selectivity was verified by analyzing cannabinoid‐free saliva samples for interferences. Linearity was assessed using calibrators at seven ascending concentration levels. Intra‐assay accuracy and precision were determined using six replicate quality control (QC) samples at low, medium, and high concentrations relative to the concentration range on the same day. Inter‐assay accuracy and precision were determined using similar QC samples three different days (three replicates per day). Repeat injections at 0‐, 4‐, and 8‐hour timepoints were used to assess autosampler stability. Dilution integrity was assessed for 10x dilutions. The lower limit of quantification (LLOQ) was selected based on accuracy of calibrator samples (lowest calibrator within ±20% of the nominal value), while the LOD was set as the lowest calibrator concentration with signal‐to‐noise greater than 3. Samples that fell above the linear quantification range were diluted appropriately and re‐analyzed.

**Table 2:** Validation parameters for oral fluid analysis of THC and CBD by LC–MS/MS

Parameter	THC	CBD
Retention time (min)	8.2	6.7
Quantifier transition (qualifier transition)	315.1 → 193.1 (315.1 → 259.1)	315.1 → 193.1 (315.1 → 259.1)
Internal standard (IS)	*d*_*3*_‐THC	*d*_*3*_‐CBD
IS quantifier transition (qualifier transition)	318.1 → 196.1 (318.1 → 123.1)	318.1 → 196.1 (318.1 → 123.1)
Specificity	No interferences found	No interferences found
Matrix effect % (*n* = 6)	79	87
LOD	1	1
LLOQ	2	6
Linearity		
*R* ^*2*^	>.996	>.997
*Linear range*	2–400	6–400
Accuracy %, intra‐assay (*n* = 6)		
*Low*	96.8	97.2
*Medium*	108.6	109.2
*High*	101.1	101.7
Accuracy %, inter‐assay (*n* = 9)		
*Low*	105.1	105.6
*Medium*	101.5	101.9
*High*	98.7	99.3
Precision %RSD, intra‐assay (n = 6)		
*Low*	10.4	10.1
*Medium*	10.5	10.8
*High*	5.4	4.7
Precision %RSD, inter‐assay (n = 9)		
*Low*	10.8	11.0
*Medium*	10.0	10.3
*High*	8.0	7.2
Autosampler stability (% 0 h timepoint)		
*4 h*	101.1	98.0
*8 h*	97.1	98.1
Dilution integrity (10x dilution; n = 6)		
*Medium QC accuracy (%)*	102.2	103.3
*Medium QC precision (%RSD)*	9.2	7.5

LOD = limit of detection. LLOQ = lower limit of quantification. N.B. For accuracy and precision, low = 10 ng/mL, medium = 100 ng/mL, and high = 400 ng/mL.

### 2.5 Interpretation of screening test results and statistical analyses

Based on the LC–MS/MS quantified concentration of THC in the corresponding oral fluid sample, each screening test result was classified as a true positive (TP), true negative (TN), false positive (FP), or false negative (FN). A *true positive* was a positive test result that was subsequently confirmed by LC–MS/MS (ie, confirmed value > confirmatory cut‐off AND positive result obtained). A *true negative* was a negative test result which was confirmed by LC–MS/MS (ie, confirmed value < confirmatory cut‐off AND negative result obtained). A *false positive* was a positive test result which was not confirmed by LC–MS/MS (ie, confirmed value < confirmatory cut‐off AND positive result obtained), while a *false negative* was a negative test result that was not confirmed by LC–MS/MS (ie, confirmed value > confirmatory cut‐off AND negative result obtained).

Based on these classifications, sensitivity [TP/(TP + FN)], specificity [TN/TN + FP)], and accuracy [(TP + TN)/(TP + TN + FP+ FN)] were calculated at a confirmatory cut‐off of 10 ng/mL THC (equivalent to the screening cut‐off for both devices). As the cut‐offs used for confirmatory analysis are in practice often lower than the screening cut‐off (typically to maximize the true positive rate and reduce the false positive rate), sensitivity, specificity, and accuracy were also calculated relative to THC cut‐offs of 2 ng/mL (THC LOQ) and 1 ng/mL (THC LOD).

THC and CBD concentrations in oral fluid were compared across the three conditions (THC, THC/CBD, and placebo) at each timepoint (10, 60, 120, and 180 minutes) using non‐parametric Friedman tests and post‐hoc Bonferroni comparisons. The level of statistical significance was set at *p* < 0.05. Area under the curve (AUC) was calculated for each individual from time 0 to the time of the last detectable THC concentration using the trapezoid method. All statistical analyses were performed in SPSS v24 (IBM Corp, Armonk, NY, USA).

## 3 RESULTS

### 3.1 Participants

Table 1 presents the characteristics and cannabis use history of the 14 healthy adults (11 males, 21–38 years old) who completed all three test sessions. Oral fluid samples (*N* = 210) were collected prior to and up to 3 hours after vaporization. A total of 165/168 DW5s and 163/168 DT5000 tests were considered valid and subsequently evaluated against LC–MS/MS quantified confirmatory THC concentrations.

### 3.2 LC–MS/MS method

The LC–MS/MS method was accurate, precise, and had LODs of 1 ng/mL for both THC and CBD, and LLOQs of 2 and 6 ng/mL for THC and CBD, respectively. Although some matrix effect was apparent, this was accounted for with the use of deuterated internal standards for both analytes. We also verified that other common phytocannabinoids that could also be present in saliva (THCA, THCV, CBN, CBDA, CBG, CBGA, and CBC) were chromatographically separated and did not interfere with CBD or THC quantification (data not shown).

### 3.3 Oral fluid cannabinoid concentrations

**Figure 1:**
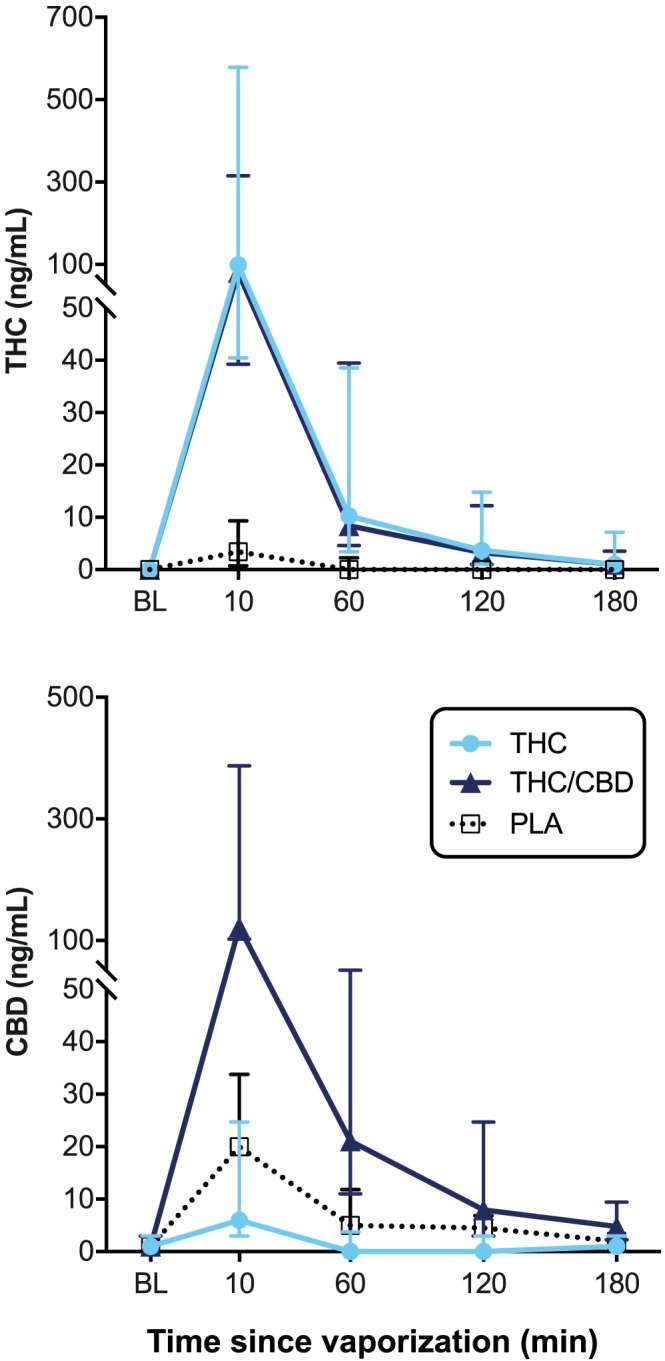
Median (Interquartile range) oral fluid THC and CBD concentrations over time as determined by confirmatory LC−MS/MS analysis following vaporization of THC‐dominant (THC), THC/CBD‐equivalent (THC/CBD), and placebo (PLA) cannabis [Colour figure can be viewed at http://wileyonlinelibrary.com]

Table 3 presents THC and CBD pharmacokinetic data for each individual, while Figure 1 shows median THC and CBD concentrations over time. All baseline THC concentrations were < LOQ with the exception of one sample with a concentration of 11.4 ng/mL THC. Because the corresponding DW5s drug screen was negative for THC and the participant reported nil cannabis use since the previous session, this test session continued as normal. All baseline CBD concentrations were also <LOQ with the exception of one sample which contained 5.5 ng/mL CBD.

**Table 3:** Maximum oral fluid cannabinoid analyte concentrations (C_max_), time to C_max_ (T_max_), concentrations at 3 hours (C3h, final sample) and area under the curve (AUC) for 14 occasional cannabis users following vaporization of THC‐dominant (THC), THC/CBD‐equivalent (THC/CBD) and placebo (PLA) cannabis

Subject	THC				THC/CBD				PLA			
	C_max_ ng/mL	T_max_ h	C_3h_ ng/mL	AUC_0‐3h_	C_max_ ng/mL	T_max_ h	C_3h_ ng/mL	AUC_0‐3h_	C_max_ ng/mL	T_max_ h	C_3h_ ng/mL	AUC_0‐3h_
**THC**												
1	203	0.17	<LOQ	210.00	39	0.17	<LOQ	39.04	8.1	0.17	<LOD	7.67
2	105.4	0.17	5.4	150.40	804.7	0.17	4.9	723.00	5.4	0.17	<LOD	6.43
3	44.4	0.17	<LOQ	43.99	39.3	0.17	<LOQ	47.20	<LOQ	0.17	<LOD	.83
4	615.5	0.17	3.7	549.90	1740.6	0.17	3.1	1577.00	12.9	0.17	<LOD	14.32
5	28.7	0.17	<LOD	25.83	91.1	0.17	<LOQ	85.80	4.6	0.17	<LOD	3.83
6	92.8	0.17	<LOD	81.73	286.2	0.17	<LOQ	258.50	<LOD		<LOD	.00
7	107.5	0.17	<LOD	104.00	14.2	0.17	<LOD	13.76	<LOD		<LOD	.00
8	19.9	0.17	<LOQ	17.50	54.6	0.17	<LOQ	50.68	2	0.17	<LOD	1.67
9	25.7	0.17	<LOQ	24.71	61.5	0.17	<LOQ	54.73	<LOD		<LOD	.00
10	1318	0.17	4.1	1207.00	6.3	0.17	<LOQ	9.18	4.9	0.17	<LOD	4.08
11	787.6	0.17	4.4	695.00	137.1	0.17	15.4	175.40	<LOQ	0.17	<LOD	.83
12	53.4	0.17	<LOQ	56.87	70.6	0.17	<LOD	71.91	2.2	0.17	<LOD	1.83
13	51.6	0.17	<LOQ	49.54	236.2	0.17	<LOQ	204.70	36.5	0.17	12.3	67.91
14	566.4	0.17	21.6	623.70	400.9	0.17	23.7	453.80	23	0.17	11.2	48.97
**CBD**												
1	17	0.17	<LOQ	28.35	117.4	0.17	8.5	130.40	33.8	0.17	<LOQ	49.00
2	9	0.17	<LOQ	18.84	1274.8	0.17	14.8	1183.00	33.8	0.17	<LOQ	51.42
3	34.1	0.17	<LOQ	36.92	124.7	0.17	9.1	176.70	15	0.17	<LOQ	30.28
4	24.5	0.17	<LOQ	31.67	2934.9	0.17	9.8	2711.00	76.3	0.17	6.2	100.90
5	<LOQ	0.17	<LOD	2.92	190.5	0.17	9.3	198.10	24.9	0.17	<LOQ	36.22
6	<LOQ	0.17	<LOD	3.42	462.8	0.17	<LOQ	431.70	<LOQ	0.17	<LOD	3.42
7	<LOQ	0.17	<LOD	3.42	29.2	0.17	<LOD	30.08	<LOQ	0.17	<LOD	6.17
8	<LOD		<LOD	.92	99.7	0.17	<LOQ	99.37	15.2	0.17	<LOD	16.33
9	<LOQ	0.17	<LOD	3.42	103.3	0.17	<LOD	95.00	<LOQ	0.17	<LOD	3.42
10	75.8	0.17	<LOD	22.00	15.3	0.17	<LOD	22.58	24.8	0.17	<LOD	21.58
11	84.9	0.17	<LOD	24.50	328	0.17	19.7	129.20	<LOQ	0.17	<LOD	3.42
12	<LOQ	0.17	<LOD	3.42	120	0.17	<LOQ	128.50	9.4	0.17	<LOD	14.50
13	<LOQ	0.17	<LOD	6.17	362	0.17	<LOQ	319.20	209	0.17	20.1	82.06
14	33.7	0.17	<LOD	13.00	925.8	0.17	47.7	354.50	87.5	0.17	12	40.88

Concentrations of oral fluid THC and CBD (Figure 1) were maximal (C_max_) at the 10‐minute post‐vaporization timepoint for all individuals and declined rapidly thereafter. The mean (range) for THC C_max_ was 287.1 (19.9–1318) ng/mL in the THC condition, 285.5 (6.3–1740.6) ng/mL in the THC/CBD condition, and 7.26 (0–36.5) ng/mL in the placebo condition. At 3 hours, the mean (range) THC concentrations were 4.3 (0–21.6) and 3.8 (0–23.7) ng/mL in the THC and THC/CBD conditions, respectively, and 1.7 (0–12.3) ng/mL in the placebo condition.

The mean (range) for CBD C_max_ was 21.21 (0–84.9) ng/mL in the THC condition, 506.3 (15.3–2934.9) ng/mL in the THC/CBD condition, and 36.7 (3–209) ng/mL in the placebo condition. At 3 hours, the mean (range) CBD concentrations were 1.4 (0–3.0) and 9.4 (0–47.7) ng/mL in the THC and THC/CBD conditions, respectively, and 3.6 (0–20.1) ng/mL in the placebo condition.

Oral fluid THC concentrations differed significantly between the three conditions at 10 minutes (χ^2^ (2) = 21.14, *p* < .001) and 60 minutes (χ^2^ (2) = 21.57, *p* < .001) but not at baseline or at the 120‐minute or 180‐minute timepoints. At 10 minutes, oral fluid THC concentrations were significantly higher in both the THC (*p* < .001) and THC/CBD (*p* < .001) conditions than in the placebo condition. At 60 minutes, THC concentrations were also significantly higher than placebo in both the THC (*p* = .001) and THC/CBD (*p* < .001) conditions. There were no significant differences between the THC and THC/CBD conditions at any timepoint.

Oral fluid CBD concentrations differed significantly between conditions at 10 minutes (χ^2^ (2) = 17.25, *p* < .001), 60 minutes (χ^2^ (2) = 23.57, *p* < .001), 120 minutes (χ^2^ (2) = 21.28, *p* < .001), and 180 minutes (χ^2^ (2) = 7.48, *p* = .024). There were no differences between groups at baseline. At 10 minutes, CBD concentrations were significantly greater in the THC/CBD condition than in the THC (*p* < .001) or placebo (*p* = .01) conditions. At 60 and 120 minutes, CBD concentrations were also significantly higher in the THC/CBD condition than in either the THC (*p* < .001) or placebo (*p* = .042) conditions. At 240 minutes, CBD concentrations were still higher in the THC/CBD condition than they were in the THC condition (*p* = .042).

Mean [range] area under the curve (AUC_0‐ > 3h_) for THC was similar in the THC/CBD (268.9 [9.2–1577] ng/mL x h) and THC conditions (274.3 [17.5–1207] ng/mL x h). Mean [range] AUC_0‐ > 3h_ for CBD was significantly higher in the THC/CBD condition (429.2 [22–6 – 2711] ng/mL x h) than in the THC (14.2 [0.9–36.9] ng/mL x h) or placebo (32.9 [3.4–100.9] ng/mL x h) conditions (*p* = .001; *p* = .001).

### 3.4 POCT device performance

**Figure 2:**
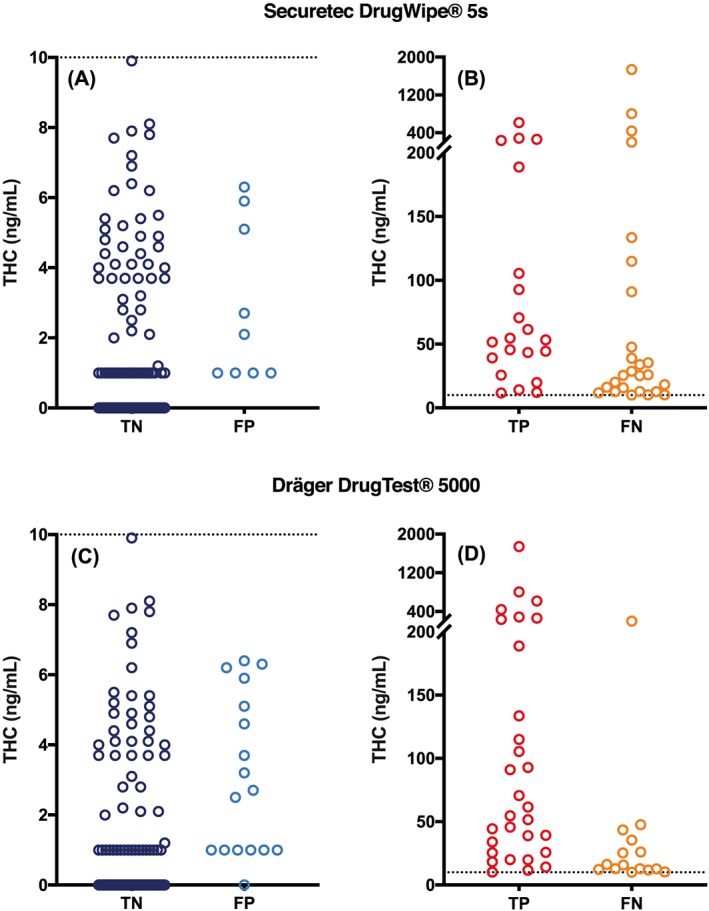
LC−MS/MS confirmed oral fluid THC concentrations corresponding to A, DrugWipe 5s true negative (TN) and false positive (FP) test results; B, DrugWipe 5s true positive (TP) and false negative (FN) test results; C, DrugTest 5000 TN and FP test results; and D, DrugTest 5000 TP and FN test results. The dotted line marks the screening cutoff (10 ng/mL) [Colour figure can be viewed at http://wileyonlinelibrary.com]

Table 4 presents the test results (TP, TN, FP, FN) for the DW5s and DT5000 and overall device performance (sensitivity, specificity, and accuracy) at a 10 ng/mL confirmatory cut‐off, while Table 5 describes these parameters when a 2 ng/mL and 1 ng/mL confirmatory cut‐offs are applied. Figure 2 shows the LC–MS/MS quantified THC concentration corresponding to each test result.

**Table 4:** Performance characteristics of the Securetec DrugWipe^®^ 5 s (DW5s) and Dräger DrugTest^®^ 5000 (DT5000) POCT devices when verified against LC–MS/MS quantified oral fluid THC concentrations using a 10 ng/mL confirmatory cut‐off

Device	Time (min)	Total *N* of Tests	TP	TN	FP	FN	Sensitivity (%)	Specificity (%)	Accuracy (%)
*DW5s*	10	41	19	12	1	9	68	92	76
	60	40	2	24	4	10	17	86	65
	120	42	0	32	3	7	‐ *	91	76
	180	42	0	41	1	0	‐ *	98	98
	Total	165	21	109	9	26	45	92	79
*DT5000*	10	39	23	12	1	3	88	92	90
	60	40	6	20	8	6	50	71	65
	120	42	1	29	6	6	14	83	71
	180	42	0	40	2	0	‐ *	95	95
	Total	163	30	101	17	15	67	86	80

POCT = Point‐of‐collection testing.

* Sensitivity could not be calculated as there were no true positives.

**Table 5:** Performance characteristics of the Securetec DrugWipe^®^ 5 s (DW5s) and Dräger DrugTest^®^ 5000 (DT5000) POCT devices when verified against LC–MS/MS quantified oral fluid THC concentrations using confirmatory cut‐offs of 2 ng/mL and 1 ng/mL

Device	Cut‐off	Time (min)	Total *N* of Tests	TP	TN	FP	FN	Sensitivity (%)	Specificity (%)	Accuracy (%)
DW5s	2 ng/mL	10	41	20	5	0	16	56	100	61
		60	40	6	12	0	22	21	100	45
		120	42	0	21	3	18	‐ *	88	50
		180	42	0	30	1	11	‐ *	97	71
		Total	165	26	68	4	67	28	94	57
	1 ng/mL	10	41	20	3	0	18	53	100	56
		60	40	6	9	0	25	19	100	38
		120	42	3	14	0	25	11	100	40
		180	42	1	21	0	20	5	100	52
		Total	165	30	47	0	88	25	100	47
DT5000	2 ng/mL	10	39	24	5	0	10	71	100	74
		60	40	13	11	1	15	46	92	60
		120	42	3	20	4	15	17	83	55
		180	42	0	29	2	11	‐ *	94	69
		Total	163	40	65	7	51	44	90	64
	1 ng/mL	10	39	24	3	0	12	67	100	69
		60	40	14	9	0	17	45	100	58
		120	42	7	14	0	21	25	100	50
		180	42	1	20	1	20	5	95	50
		Total	163	46	46	1	70	40	98	56

POCT = Point‐of‐collection testing.

* Sensitivity could not be calculated as there were no true positives.

### 3.5 DrugWipe 5 s

A total of 165 DW5s test results involving four different time points were evaluated against LC–MS/MS verified oral fluid THC concentrations. With a 10 ng/mL confirmatory cut‐off applied (Table 4), overall sensitivity, specificity, and accuracy were calculated as 45%, 92%, and 79%. Of the 30 test results that were positive, 9 false positives were detected with corresponding oral fluid THC concentrations ranging from 1.0 to 6.3 ng/mL. Of the 135 test results that were negative, 26 false negatives were detected, with corresponding oral fluid THC concentrations ranging from 10.1 to 1740 ng/mL. The occurrence of both false positives and false negatives was greatest at the 60‐minute timepoint. As Table 5 shows, fewer false positives and more false negatives were observed with confirmatory cut‐offs of 2 ng/mL and 1 ng/mL. Overall accuracy was greatest with a 10 ng/mL confirmatory cut‐off applied.

### 3.6 DrugTest 5000

A total of 163 DT5000 test results involving four different time points were evaluated relative to LC–MS/MS verified oral fluid THC concentrations. At a 10 ng/mL confirmatory cut‐off (Table 4), overall sensitivity, specificity, and accuracy were calculated as 67%, 86%, and 80%. Of the 47 test results that were positive, 17 false positives were detected with corresponding oral fluid THC concentrations ranging from 0 to 6.4 ng/mL. Of the 116 test results that were negative, 15 false negatives were detected, with corresponding oral fluid THC concentrations ranging from 10.1 to 203 ng/mL. As with the DW5s, the incidence of false positives and false negatives were greatest at the 60‐minute timepoint. Applying a confirmatory cut‐off of 2 ng/mL or 1 ng/mL decreased the number of false positives but substantially increased the number of false negatives (Table 5). Overall accuracy was highest with a 10 ng/mL confirmatory cut‐off.

## 4 DISCUSSION

The present study was designed to provide insights into the accuracy and reliability of two commonly used POCT devices. We assessed the performance of the DW5s and DT5000 devices by comparing observed test results against confirmatory LC–MS/MS quantified oral fluid THC and CBD concentrations at various timepoints following controlled laboratory vaporization of three different cannabis types (placebo, THC‐dominant, and THC/CBD‐equivalent) using a within‐subjects, crossover design.

Overall, our data confirm that oral fluid THC is a good indicator of very recent cannabis use.^20^, ^24^, ^34^, ^35^, ^36^ As with previous studies,^24^, ^25^, ^34^, ^36^, ^37^ oral fluid cannabinoid concentrations were maximal at the time point closest to vaporization (10 minutes) and declined rapidly thereafter. The high inter‐ and intra‐individual variability in peak THC concentrations that we observed here is consistent with previous studies involving smoked or vaporized cannabis. For example, Toennes et al^38^ reported peak oral fluid THC concentrations of 387–71,147 ng/mL in chronic cannabis users 0.08 hours after smoking 500 μg/kg THC (equivalent to 37.5 mg THC for a 75 kg individual),^38^ while Swortwood et al^24^ observed peak THC concentrations ranging from 68.6–7373 ng/mL at 0.17 hours after vaporization of cannabis containing ~50.6 mg THC. In the present study, peak THC concentrations ranged from 19 to 1318 ng/mL in the THC condition and 6.3 to 1740.6 ng/mL in the THC/CBD condition following vaporization of cannabis containing ~13.75 mg THC. Maximum CBD concentrations varied between 15.3 and 3924 ng/mL in the THC/CBD condition with CBD concentrations, as expected, relatively low in the other two conditions.

The extreme variability in oral fluid cannabinoid concentrations observed here may reflect variability in inhalation topography. While we dictated duration of inhalation and breath holding as well as the amount of time between inhalations, we were unable to control inhalation volume or depth . Notably, Huestis et al^39^ also reported significant variability in oral fluid cannabinoid concentrations when using a controlled inhalation procedure. Other factors that may influence this include dry mouth (a common side effect of cannabis), oral fluid collection volume and differences in oral fluid composition and flow rate.^40^

THC concentrations in oral fluid were very similar in the THC‐dominant and THC/CBD equivalent cannabis conditions. A previous study^41^ similarly found no significant difference in mean THC C_max_ or AUC following sublingual administration of purified THC (25 mg) alone or in combination with CBD (25 mg). While these results suggest that the presence of CBD has minimal − if any − effects on the detection and quantification of THC in oral fluid, we acknowledge that only equivalent CBD and THC concentrations were examined here. In reality, cannabis chemovars and extracts may contain far higher ratios of CBD to THC. For example, the so‐called ‘light cannabis' varieties that are legally available through much of the EU must contain less than 0.2% THC but may contain up to 40% CBD.^29^

In a recent study, oral fluid THC concentrations reached 21.5 ng/mL at 30 minutes after participants smoked 1 g of ‘light cannabis' containing 5.8% CBD (~ 58 mg) and 0.16% THC (~ 1.6 mg).^29^ This matches and exceeds the observed THC C_max_ for several individuals in the present study and is well above the DW5s and DT5000 detection limit of 10 ng/mL. Consistent with this, it is notable that two participants in the present study had oral fluid THC concentrations >10 ng/mL after vaporizing placebo cannabis containing only minor amounts (< 1%) of THC. Taken together, these data suggest that consumption of high CBD cannabis with very low THC content may still result in a positive DW5s or DT5000 test result, even in the absence of any driving impairment.^32^ This raises important questions around the validity of the MDT program and other DUIC programs involving POCT for oral fluid THC.

Both the DW5s and DT5000 showed high specificity, which is the proportion of confirmed negatives in cases where the POCT test result was negative. Sensitivity, however, was generally very poor. This reflects the high incidence of false negatives, where oral fluid samples corresponding to negative test results were found to have THC concentrations above the device screening cut‐off (ie, > 10 ng/mL). The false positive rate was also concerning: 9 false positives were detected by the DW5s, and 17 by the DT5000. Only the DT5000 met DRUID criterion^9^ for accuracy, which is the ability of a test to correctly discriminate between positive and negative cases. Sensitivity was highest at 10 minutes, when the incidence of positive test results was highest. Accuracy was highest for both devices at 180 minutes, when all samples were found to be true negatives, but this is something of an artefact given that sensitivity at this time point could not be computed. Accuracy was highest with a confirmatory cut‐off of 10 ng/mL, and decreased progressively with lower cut‐offs due to the substantial increase in false negatives.

These data are consistent with previous studies which have collectively reported DT5000 sensitivity, specificity and accuracy as 49.5%–100%, 55%–90%, and 55%–86.4%.^5^, ^9^, ^14^, ^16^, ^17^, ^18^, ^19^, ^20^ One controlled laboratory study^20^ reported considerably better DT5000 performance (>80% specificity, sensitivity, and accuracy) than we observed here. In that study, however, samples were collected up to 72 hours after cannabis administration and therefore included a much larger proportion of samples with very low THC concentrations that were classified as true negatives. Another study^19^ reported DT5000 specificity and accuracy similar to that observed here, but with greater sensitivity than reported here (92.7% vs 67%). This discrepancy is due to the difference in the ratio of false negative to true positive results: a total of 38 true positives and 3 false negatives were detected out of 66 samples, whereas in the present study, 30 true positives and 15 false negatives were detected out of 163 samples. The higher incidence of true positives^19^ reflects the much higher dose of THC (54 mg) than what was used here (13.75 mg) which produced much higher oral fluid THC concentrations. However, the percentage of false positives (9.1%) was very similar to the present study (10.4%).

The present results are also consistent with those of Wille et al,^15^ who used a previous version of the DW5s (15 ng/mL cut‐off) and a confirmatory cut‐off of 10 ng/mL. Sensitivity, specificity and accuracy were reported as 51%, 100%, and 68%, whereas in the present study they were 45%, 92%, and 79%. While Wille et al^15^ reported 0 false positives out of 79 tests, testing was only done immediately and at 80 minutes after vaporization of 300 μg/kg THC, leading to high concentrations of oral fluid THC – the lowest being 34 ng/g – and thus minimizing the possibility of obtaining a false positive result. In the present study, 9 false positives (5.4% of overall results) were detected with a 10 ng/mL confirmatory cut‐off, reflecting the lower dose that we administered. In both studies, DW5s sensitivity was best immediately after vaporization when oral fluid THC concentrations were maximal.

While the short detection window for THC is a key benefit of the POCT approach, the erratic distribution of THC in oral fluid, and the magnitude of intra‐ and inter‐individual variability following standardized cannabis administration, may preclude its use as a meaningful marker of acute intoxication or impairment. We recently demonstrated impaired driving performance and reduced confidence in driving ability in these same 14 participants at both 30 and 210 minutes following vaporization.^32^ However, at 180 minutes, there were no true positive test results in the present study and THC concentrations were typically below the LOQ. Consistent with this, a controlled laboratory study by Ramaekers et al^42^ found only a weak relationship between oral fluid THC concentrations and magnitude of impairment on a range of driving‐related cognitive tasks following smoked cannabis. Moreover, oral fluid THC concentrations can exceed 10 ng/mL (ie, the DW5s and DT5000 screening cut‐offs) following passive exposure to cannabis smoke^43^, ^44^ or consumption of high CBD cannabis with negligible THC content.^29^ It is therefore possible for an individual who has not actually consumed cannabis to test positive for cannabis with the two POCT devices examined here. These findings offer some support to concerns around the validity of the MDT program.^45^ Given the widespread and increasing use of POCT as a method for detecting DUIC, it is essential that these limitations are considered.

## 5 CONCLUSIONS

Here we have compared, for the first time, cannabinoid concentrations in oral fluid following controlled administration of THC‐dominant, THC/CBD equivalent, and placebo cannabis. There were few differences in confirmed oral fluid THC concentrations between the two active cannabis conditions, suggesting that CBD has little effect on oral fluid THC concentrations when the two compounds are vaporized in a 1:1 ratio. It may not be possible to generalize these results to other routes of administration and to cannabis or cannabinoid products containing higher CBD to THC ratios. We also evaluated the performance of the DW5s and DT5000 POCT devices that are widely used to detect DUIC. Both devices performed acceptably when oral fluid THC concentrations were well above or below the screening cut‐off, but neither device exhibited >80% sensitivity, specificity, and accuracy. A considerable number of false positive and false negative results were observed. While these devices are useful tools for detecting recent cannabis use, confirmatory testing is absolutely necessary and of the utmost importance. This is especially important in contexts (eg, DUIC) where positive tests results may lead to criminal convictions.
